# Burden of disease attributed to ambient air pollution in Thailand: A GIS-based approach

**DOI:** 10.1371/journal.pone.0189909

**Published:** 2017-12-21

**Authors:** Chayut Pinichka, Nuttapat Makka, Decharut Sukkumnoed, Suwat Chariyalertsak, Puchong Inchai, Kanitta Bundhamcharoen

**Affiliations:** 1 International Health Policy Program, Ministry of Public Health, Nonthaburi, Thailand; 2 Health Systems Research Institute, Research and Development Program on Healthy Public Policy and Health Impact Assessment, Nonthaburi, Thailand; 3 Research Institute for Health Sciences, Chiang Mai University, Sriphum, Muang Chiang Mai, Thailand; University of Tennessee Health Science Center, UNITED STATES

## Abstract

**Background:**

Growing urbanisation and population requiring enhanced electricity generation as well as the increasing numbers of fossil fuel in Thailand pose important challenges to air quality management which impacts on the health of the population. Mortality attributed to ambient air pollution is one of the sustainable development goals (SDGs). We estimated the spatial pattern of mortality burden attributable to selected ambient air pollution in 2009 based on the empirical evidence in Thailand.

**Methods:**

We estimated the burden of disease attributable to ambient air pollution based on the comparative risk assessment (CRA) framework developed by the World Health Organization (WHO) and the Global Burden of Disease study (GBD). We integrated geographical information systems (GIS)-based exposure assessments into spatial interpolation models to estimate ambient air pollutant concentrations, the population distribution of exposure and the concentration-response (CR) relationship to quantify ambient air pollution exposure and associated mortality. We obtained air quality data from the Pollution Control Department (PCD) of Thailand surface air pollution monitoring network sources and estimated the CR relationship between relative risk (RR) and concentration of air pollutants from the epidemiological literature.

**Results:**

We estimated 650–38,410 ambient air pollution-related fatalities and 160–5,982 fatalities that could have been avoided with a 20 reduction in ambient air pollutant concentrations. The summation of population-attributable fraction (PAF) of the disease burden for all-causes mortality in adults due to NO_2_ and PM_2.5_ were the highest among all air pollutants at 10% and 7.5%, respectively. The PAF summation of PM_2.5_ for lung cancer and cardiovascular disease were 16.8% and 14.6% respectively and the PAF summations of mortality attributable to PM_10_ was 3.4% for all-causes mortality, 1.7% for respiratory and 3.8% for cardiovascular mortality, while the PAF summation of mortality attributable to NO_2_ was 7.8% for respiratory mortality in Thailand.

**Conclusion:**

Mortality due to ambient air pollution in Thailand varies across the country. Geographical distribution estimates can identify high exposure areas for planners and policy-makers. Our results suggest that the benefits of a 20% reduction in ambient air pollution concentration could prevent up to 25% of avoidable fatalities each year in all-causes, respiratory and cardiovascular categories. Furthermore, our findings can provide guidelines for future epidemiological investigations and policy decisions to achieve the SDGs.

## Introduction

Air pollution is a major global concern. Epidemiological studies have shown that exposure to ambient air pollution leads to adverse health effects, including increases in mortality and morbidity from cardiovascular and respiratory diseases [1–3]. Mortality attributed to ambient air pollution is identified as an indicator of the sustainable development goals (SDGs) [4]. Globally, ambient particulate matter pollution accounted for 4.2 million deaths and 103 million healthy life-years lost in 2015, representing 7.6% of total global mortality and making it the fifth-ranked global risk factor in the Global Burden of Diseases Study 2015 (GBD 2015) [5, 6].

Quantitative analyses of how different risk factors contribute to the overall disease burden provide critical information for health policymaking and priority-setting. The comparative risk assessment (CRA) approach developed by the World Health Organisation (WHO) and the GBD provides a framework for population risk assessment and comparison across risks at both global and national levels [6, 7].

Increasing urbanisation, industrialised area, traffic congestion, forest fires and agricultural burning contribute to escalating air pollution in Thailand [8, 9]. The pattern of exposure has differed across various areas with the specific characteristics of pollutant sources; furthermore, the effects from air pollution may vary at the subnational level, especially in urban area[10]. GBD [6, 7, 11] and previous CRA study in Thailand [12] estimated the disease burden attributable to ambient air pollution at national and regional scale but did not provide distribution at sub-national levels across the country. Other pollutants; such as, coarser particle matter (PM_10_) and nitrogen dioxide (NO_2_), might also be important to quantify the public health impact as per the GBD recommendation [5].

Over several decades, Thailand has developed an extensive air quality monitoring network with the aim of providing up-to-date empirical information on ambient air pollutants (i.e. PM_2.5_, PM_10_ and NO_2_) [13]. Spatial variability of air pollution concentrations from local air quality network provides country-specific information to investigate the magnitude and distribution of the public health impact for PM_2.5_, PM_10_ and NO_2_. Geographical information systems (GIS) and spatial analysis have been used to estimate the distribution of ambient air pollution exposure at unknown locations based on empirical data in many environmental epidemiology studies [14–16]. This approach can improve estimated exposure distribution at the national, sub-national and/or specific levels [17], and also provide valuable information for policy-makers to improve air quality and health benefits in specific locations.

This study aimed to quantify the magnitude and geographical distribution of disease burden in terms of mortality attributable to ambient air-pollutant exposure based on available and observed data from air monitoring measurements. We utilised GIS to explore the spatial variability of air pollution exposure, and adopted the CRA method developed by the WHO, the GBD [18] and others [19, 20] to quantify mortality attributable to ambient air pollution.

## Methods

### Overall approach to estimate the burden attributable to ambient air pollution

**Fig 1:**
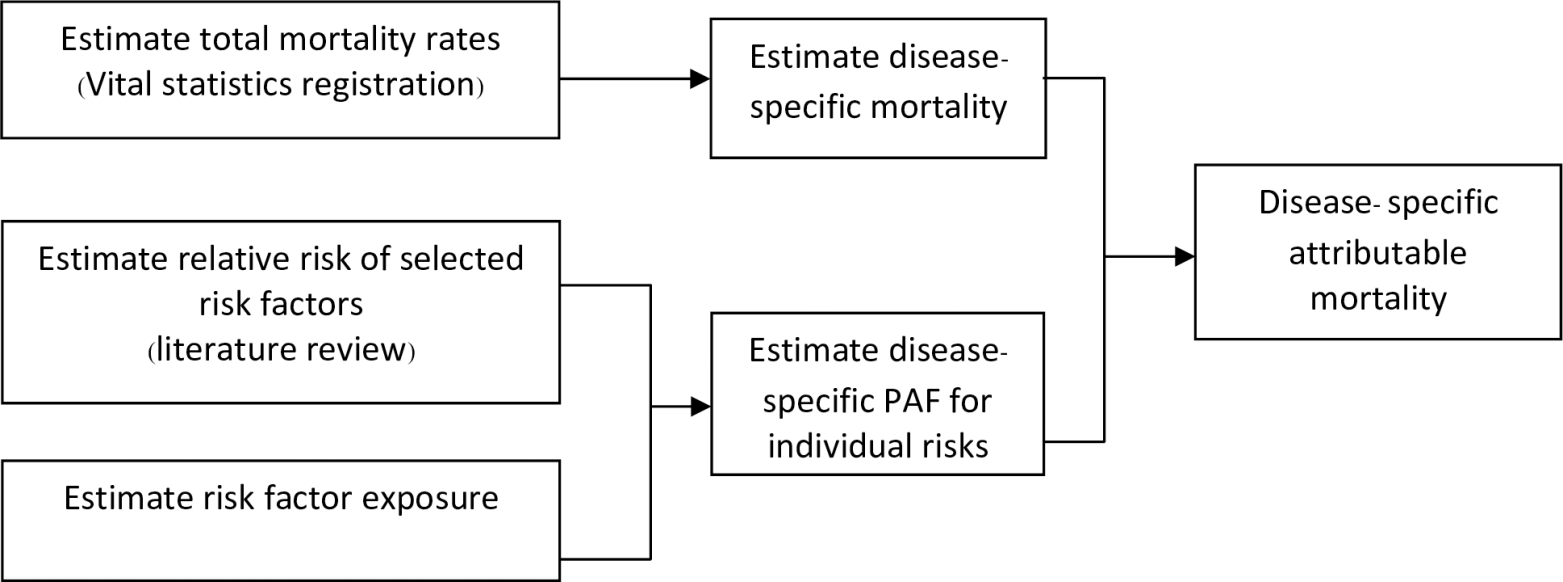
Methodology scheme of the comparative risk assessment.

We employed the CRA framework which is defined as the systematic evaluation of the changes in the population health and ranking the different factors that contribute to the specific outcome to quantify the burden of disease attributable to ambient air pollution [21]. The general framework and its components are presented in Fig 1. Each component of the estimation is described as follows:

- Ambient air pollution exposure
- Population distribution of exposure (Pe)
- Relative risks and concentration–response relationships
- Attributable mortality due to ambient air pollution

### Ambient air pollutants exposure

**Fig 2:**
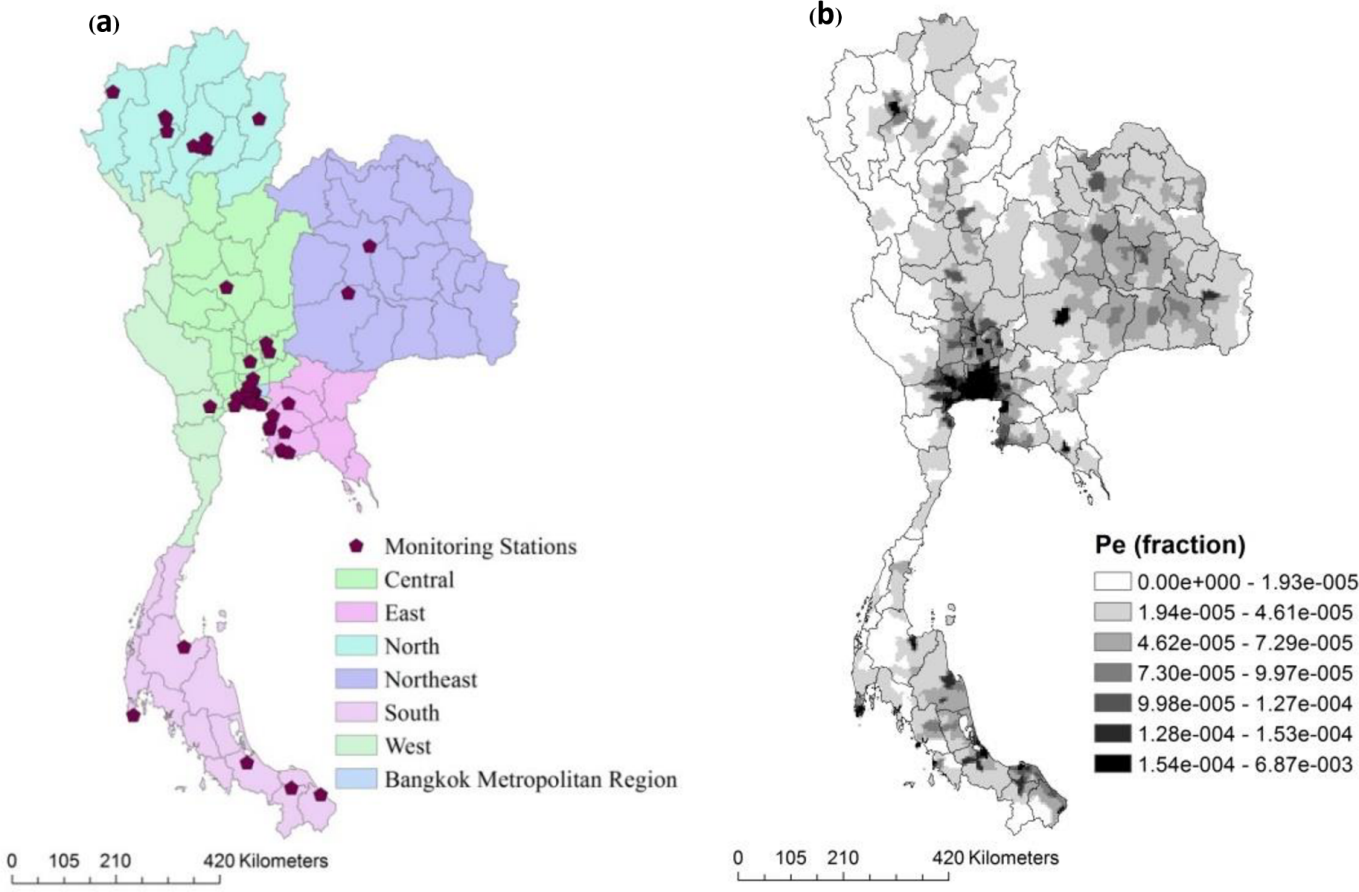
Data sources for ambient air pollution exposure. (a) Area of the study and location of surface monitoring stations network and (b) geographic pattern of population distribution of exposure (Pe).

We estimated the exposure to three main ambient air pollutants, i.e. PM_2.5_, PM_10_, and NO_2_. We obtained air pollution data from the annual report on the state of air quality in Thailand under the enhancement and conservation of the National Environmental Quality Act of 1992[22], which falls under the responsibility of the Pollution Control Department, Ministry of Natural Resources and Environment. This department has 54 monitoring stations located in all six geographical regions of Thailand as (10, 2, 8, 1, 28 and 3 stations in Northern, Northeastern, Eastern, Western, Central, and Southern regions, respectively) and extensively monitors ambient air pollutants (Fig 2). All PM was reported in micrograms per cubic metre (ugm-3). The NO_2_ concentrations was measured in parts per billion (PPB) and converted into micrograms per cubic metre using a conversion factor of 1.88 (at 25°C and 1013 millibars) for the calculated concentration response coefficients.

Many studies have indicated that the health effects of PM_2.5_ are more harmful than PM_10_[23–27] with their concentrations highly correlated[28]. The PM_10_ correlation was also very high with NO_2_ but low with SO_2_[29]. PM_10_ was reported by all existing air quality network in Thailand whereas only a few stations reported PM_2.5_ concentrations, making analysis at the country level very difficult. Since PM_2.5_ is a component of PM_10_, it is possible to estimate PM_2.5_ from PM_10_ data based on the typical relationships between pollutants, as PM_2.5_ can be treated as a fixed weight fraction of PM_10_. We decided to convert PM_10_ to PM_2.5_ for stations without PM_2.5_ readings using the ratio of PM_2.5_ to PM_10_ based on the literature review. Many studies [30–32] have reported PM_2.5_ and PM_10_ ratios in the range of 0.35–0.7, and a local study in Thailand reported ratios of about 0.5 in Bangkok[33] and clearly stated that daily PM_2.5_ and PM_10_ concentrations were highly correlated (r ≥ 0.85)[33]. From the literature review, we decided to use the ratios for PM_2.5_ and PM_10_ from the local study in Thailand[33] which was similar to the WHO global analysis of disease burden due to outdoor air pollution in developing countries [19].

To estimate the exposure level of air pollutants across geographical area, we used inverse distance weighted (IDW) interpolation method [34–39] to estimate the spatiotemporal distribution of ambient air concentrations based on empirical data from the air quality monitoring stations across Thailand and a grid consisting of 40767 cells (3×3 km^2^ resolution). For cross-validation evaluation, comparisons of predicted values to observed values were essential information about the quality of the model [40, 41] using all existing data to estimate the trend and model autocorrelation. This removed one or more data locations and predicted their associated data using information from the other locations. We then assessed the accuracy of the model using the square root of the mean for the squared prediction errors (RMSE) based on the predicted and actual values at the existing point. In addition, we obtained cross-validation correlations using the squared Pearson correlation between the measured values at known-point observations and the spatial model predictions.

To quantify the different levels of exposure to ambient air pollution for estimation of avoidable disease burden, we used a 20% reduction in ambient air pollutant as a reasonable proportion of reduction in air pollutant level from the Mexico City Air Quality Management Team [42] for possible suggestion scenarios.

### Population distribution of exposure (Pe)

To quantify the Pe, we acquired population data from the 2000 Gridded Population of the World, Version 3 (GPWv3), generated by the SEDAC (Socioeconomic Data and Applications Center) project at Columbia University [43]. This dataset was estimated from the human population from national and subnational input sources (usually administrative sources) of varying resolutions into regular latitude-longitude grids at a resolution of 2.5 arc-minute grid cells (or ~5 km at the equator). Pe was estimated as the proportion of the population by selected age groups counted in the grid divided by the total population in Thailand. We used population fractions from the Department of Provincial Administration, Thailand [44]. The total population of Thailand in 2009 was about 63 million, spread over an area of 514,000 square kilometres. We assumed the proportions of population located within each grid to have been exposed to the same pollutant concentrations in the grid cells. Pe value are presented in Fig 2. The minimum Pe per grid was zero, while the maximum was 6.9 × 10^−3^. The Pe(s) for grids in Bangkok and the vicinity were relatively high compared to other regions defined in Fig 2, reflecting higher population densities.

### Relative risks and concentration–response relationships

Air pollutants such as PM_10_, PM_2.5_, and NO_2_ can cause a variety of detrimental public health effects including cardiovascular disease (CVD), respiratory disease, and lung cancer [3, 33, 45, 46]. Relative risk (RR) is commonly used to represent the results of exposure–response functions. This study estimated health impact associated with ambient air pollution using exposure to the risk of mortality based on the relationship between RR, concentration–response coefficient and ambient air pollution concentrations [19–21, 47]. The health impact function was defined as follows:

RR=exp(β×ΔX) (1)

where β is the concentration–response coefficient (CR), as the slope of the log-linear relationship between ambient air pollution concentrations and mortality, and x–x_0_ or ΔX is the concentration change from baseline conditions or natural background concentration. We assumed natural background concentrations of 10 μgm^-3^ and 3 μgm^-3^ for PM_10_ and PM_2.5_, respectively, based on the WHO environmental burden of disease (EBD) study[19]. For NO_2_, we assumed no background concentrations (zero concentrations) for Thailand. Table 1 summarises estimations on the RRs based on the health impact function.

**Table 1:** Summary of relative risks selected to estimate the PAF of ambient air pollution in Thailand.

Pollutant	Health end-point	Types	Relative risk	CR^a^	Age group (years)	References
**PM**_**10**_	All-cause mortality^b^	Short-term	1.004 per 10 μgm^-3^	0.0004	All ages	[9]
	Respiratory mortality^c^	Short-term	1.004 per 10 μgm^-3^	0.0004	All ages	[9]
	Cardiovascular mortality^d^	Short-term	1.002 per 10 μgm^-3^	0.0002	All ages	[9]
**PM**_**2.5**_	All-cause mortality^b^	Long-term	1.06 per 10 μgm^-3^	0.006	Age >30	[3]
	Lung cancer mortality^e^	Long-term	1.14 per 10 μgm^-3^	0.013	Age >30	
	Cardiovascular mortality^d^	Long-term	1.12 per 10 μgm^-3^	0.011	Age >30	[49]
**NO**_**2**_	All-cause mortality^b^	Short-term	1.04 per 10 μgm^-3^	0.007	All ages	[50]
	Respiratory mortality^c^	Short-term	1.03 per 10 μgm^-3^	0.005	All ages	

^a^ Concentration–response coefficient.

^b^ All-cause mortality excluded deaths attributed to external causes (ICD-10 codes V01–Y89).

^c^ Respiratory mortality refers to ICD-10 code: J00-99.

^d^ Cardiovascular mortality refers to ICD-10 code: I00-99

^e^ Lung cancer mortality refers to ICD-10 codes C34.

We selected RR for short- and long-term effects based on available local study, systematic reviews and meta-analysis, as well as recommendations from previous EBD studies of ambient air pollutants. The chosen health outcomes were grouped following ICD-10 classification for all-causes mortality, except for deaths attributed to external causes (ICD-10: V01–Y89), cardiovascular disease (ICD-10: I00–I99), and respiratory disease (ICD-10: J00–J99). For PM_10_, we selected RR for all-causes mortality, respiratory and cardiovascular outcome based on available local study in Thailand [9]. The health outcomes of PM_2.5_ included all-causes mortality, cardiopulmonary mortality and lung cancer mortality in the population aged > 30 years, all due to long-term exposure to PM_2.5_, using annual average concentration as the exposure indicator [3]. Due to a lack of RR information in Thailand, this study used RR information for NO_2_ based on the systematic review and meta-analysis of 23 long-term studies on a global scale, published from 2004 to 2013, evaluating the relationship between NO_2_ and mortality outcome [48].

### Attributable mortality due to ambient air pollution

To investigate the magnitude of the disease burden attributable to ambient air pollution and mortality associated with ambient air pollution, exposure is expressed as the fraction of disease or death attributable to the risk factor in a population and referred to as the population-attributable fraction (PAF) [18, 21]. The PAF has long been used to estimate the proportion reduction of burden that can be attributed to specified risk factors [51, 52]. The exposed population may be divided into multiple categories based on the level or length of exposure, each with its own RR. With multiple (n) exposure categories, the PAF is given by the following generalised equation [18]:

$$PAF=\frac{\sum_{i=1}^{n}Pe_{i}(RR_{i}-1)}{\sum_{i=1}^{n}Pe_{i}(RR_{i}-1)+1}$$ (2)

PAF = proportion of disease burden attributable to ambient air pollution
Pe_i_ = proportion estimates of the population in exposure category i, including the unexposed
RR_i_ = relative risk (magnitude of the association between ambient air pollution and disease) in exposure category “i”, compared to the reference level

We calculated PAF using Eq 3 and performed GIS raster algebra analysis using the raster resampling technique for different resolutions. The coarse grid (Pe) served as the basis for our estimate of the total burden of disease across Thailand. To calculate the expected number of mortality cases due to ambient air pollution exposure (E), we applied PAF to the number of mortalities as the following equation;

E=PAF×N (3)

E = expected number of deaths due to ambient air pollution
N = baseline number of deaths for each disease outcome

The number of disease specific deaths was obtained from the Thai Burden of Disease (BOD) study [53] conducted every five year to provide burden of disease information to setting national health planning priorities. The BOD study estimated age-, sex-, and cause-specific mortality by verifying cause of death from the national vital registration with a nation-wide verbal autopsy (VA) study [53–55]. The VA study was conducted in 2005 based on a sample of 3,316 in-hospital and 6,328 outside-hospital deaths from 28 selected districts in nine provinces [56]. Completeness adjustment of the vital registration was based on the mid census Survey of Population Change (SPC) conducted by the National Statistical Office [57, 58].

## Results

### Ambient air pollution concentrations in Thailand

**Fig 3:**
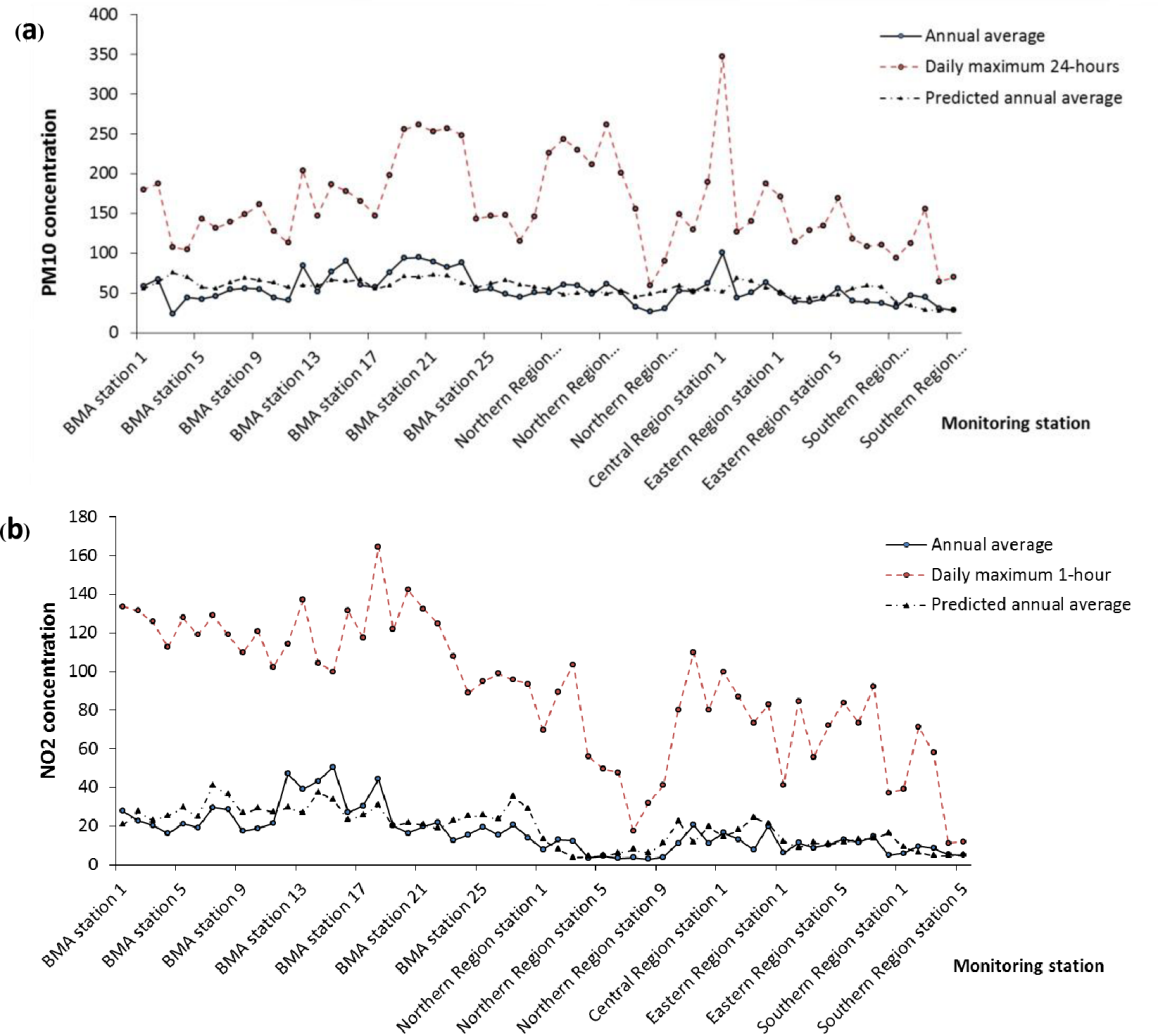
Annual average and predicted concentrations (1997–2009) by monitoring stations. (a) PM_10_ and (b) NO_2_.

Table 2 shows the statistics of average change in concentration (ΔX) values and statistical summary of model performance (best fit) corresponding to a spatial interpolation model for PM_10_, PM_2.5_, and NO_2_ from spatial interpolation based on surface monitoring measurements across Thailand. Fig 3 indicates the cross validation between the measured values at the monitoring stations and the model predictions. This study determined the average ΔX to be 41.7 μgm^-3^ (95%CI: 41.6–-41.75), 22.8 μgm^-3^ (95%CI: 22.81–22.87), 12.05 ppb (95%CI: 12–12.1), and 2.95 ppb (95%CI: 2.93–2.96), and the maximum concentrations were about 84.1 μgm^-3^, 44.1 μgm^-3^ and 48.03 ppb for PM_10_, PM_2.5_ and NO_2_, respectively (Table 2).

**Table 2:** Summary of statistical significance of the air quality data and spatial interpolation models.

Statistics measured	Pollutants					
	PM_10_ ^a^	PM_10_ model	PM_2.5_ ^a^^,^ ^b^	PM_2.5_ model	NO_2_ ^a^	NO_2_ model
**Concentrations**	54.5	41.7	27.2	22.8	17.1	12.1
**(95% CI)**	(49.6–59.3)	(41.6–41.8)	(24.8–29.7)	(22.7–22.9)	(14.1–20.1)	(12–12.1)
**Minimum**	23.5	16.9	11.7	10.4	3	3
**Maximum**	100.7	84.1	50.4	44.1	50.6	48.03
**SD**	18.4	6.97	9.2	3.5	11.3	5.2
**Correlation** ^**c**^ **(r)**	*0.44 16.7		*0.44 8.41		*0.75 7.8	
**RMSE**						

Note.

^a^ Unit: μgm^-3^ for PM, ppb for NO_2._

^b^ PM_2.5_/PM_10_ ratio = 0.5.

^**c**^ Correlation coefficients refers to measured vs. modelled concentrations.

* Correlation is significant at the 0.01 level (two-tailed).

SD: standard deviation; RMSE: root-mean-square error.

We calculated coefficients of correlation between the model predictions and the measured values at the monitoring stations. The Pearson correlation coefficients between best-fit models and actual concentrations for PM_10_, PM_2.5_ and NO_2_ were 0.44 (95%CI: 0.2–0.7), 0.75 (95%CI: 0.7–0.8), and 0.63 (95%CI: 0.5–0.75), respectively, which were statistically significant for measured ambient air concentrations (*p*-value <0.01).

**Fig 4:**
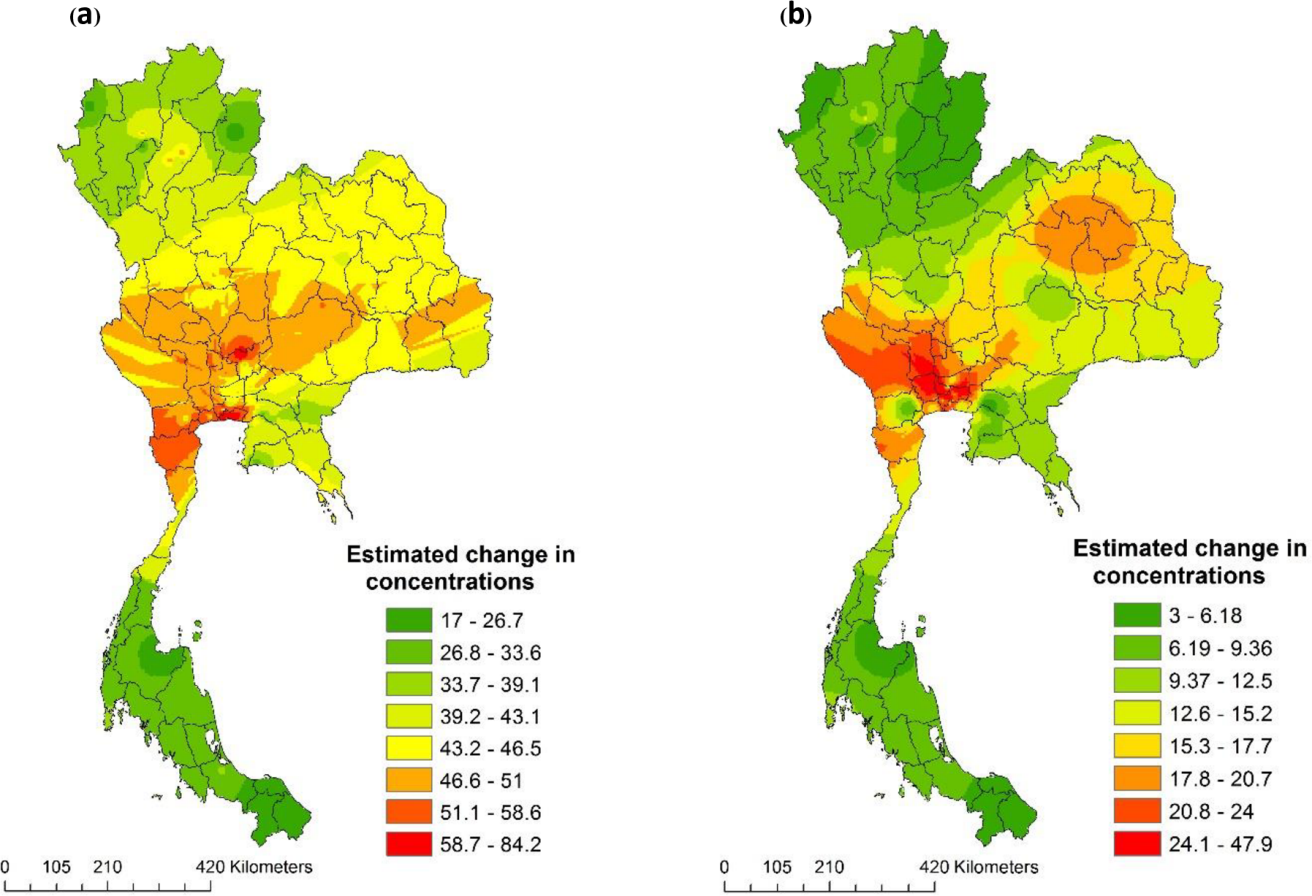
Spatial interpolation of change in concentrations (ΔX) of Thailand 2009. (a) PM10 (μgm^-3^), (b) NO2 (ppb) and (c) PM2.5 (μgm^-3^).

Fig 4 visualises the geographical distribution of annual mean estimate for exposure to ambient air pollutants across the study period. The exposure estimated for PM_10_, PM_2.5_, NO_2_ concentrations appeared in a range from 16.9 μgm^-3^ to 84.1 μgm^-3^, 10.4 μgm^-3^ to 44.1 μgm^-3^ and 3 ppb to 48 ppb, respectively, indicating that the Bangkok Metropolitan Region was more polluted than other regions in Thailand.

### Health impact and the population attributable fraction (PAF)

The health impact of ambient air pollutants was based on the relationship between change in concentrations (ΔX) and RR as described in Eq 1. Table 2 presented the estimation of RR in each pollutant and health outcome. The spatial distribution of RR across the country indicated a range from 1.01 to 1.35, depending on air pollutants and health outcome. Subsequently, this study determined the summation of PAF grids based on Eq 2 for each pollutant in Thailand in 2009, as shown in Table 3.

**Table 3:** Relative risk and population-attributable fractions (PAFs) based on level of ambient air pollutants in Thailand, 2009.

Pollutants	Health end-point (mortality)	RR				PAF (fraction)		
		Mean	SD	Max	Min	Summation	Mean (per grid)	95%CI for mean
**PM**_**10**_	All-cause	1.03	0.005	1.05	1.00	0.017	6.96E-07	6.7E-07–7.3E-07
	Respiratory	1.03	0.005	1.05	1.00	0.017	6.96E-07	6.7E-07–7.3E-07
	Cardiovascular	1.04	0.006	1.07	1.01	0.038	1.6E-06	1.5E-06–1.7E-06
**PM**_**2.5**_	All-cause	1.14	0.02	1.29	1.06	0.076	3.31E-06	3.17E-06–3.45E-06
	Lung cancer	1.35	0.06	1.80	1.15	0.169	8.16E-06	7.8E-06–8.5E-06
	Cardiovascular	1.3	0.05	1.65	1.13	0.146	6.9E-06	6.6E-06–7.2E-06
**NO**_**2**_	All-cause	1.01	0.004	1.03	1.00	0.010	4E-07	3.7E-07–4.2E-07
	Respiratory	1.037	0.02	1.16	1.00	0.025	1.03E-06	0.95E-06–1.1E-06

**Fig 5:**
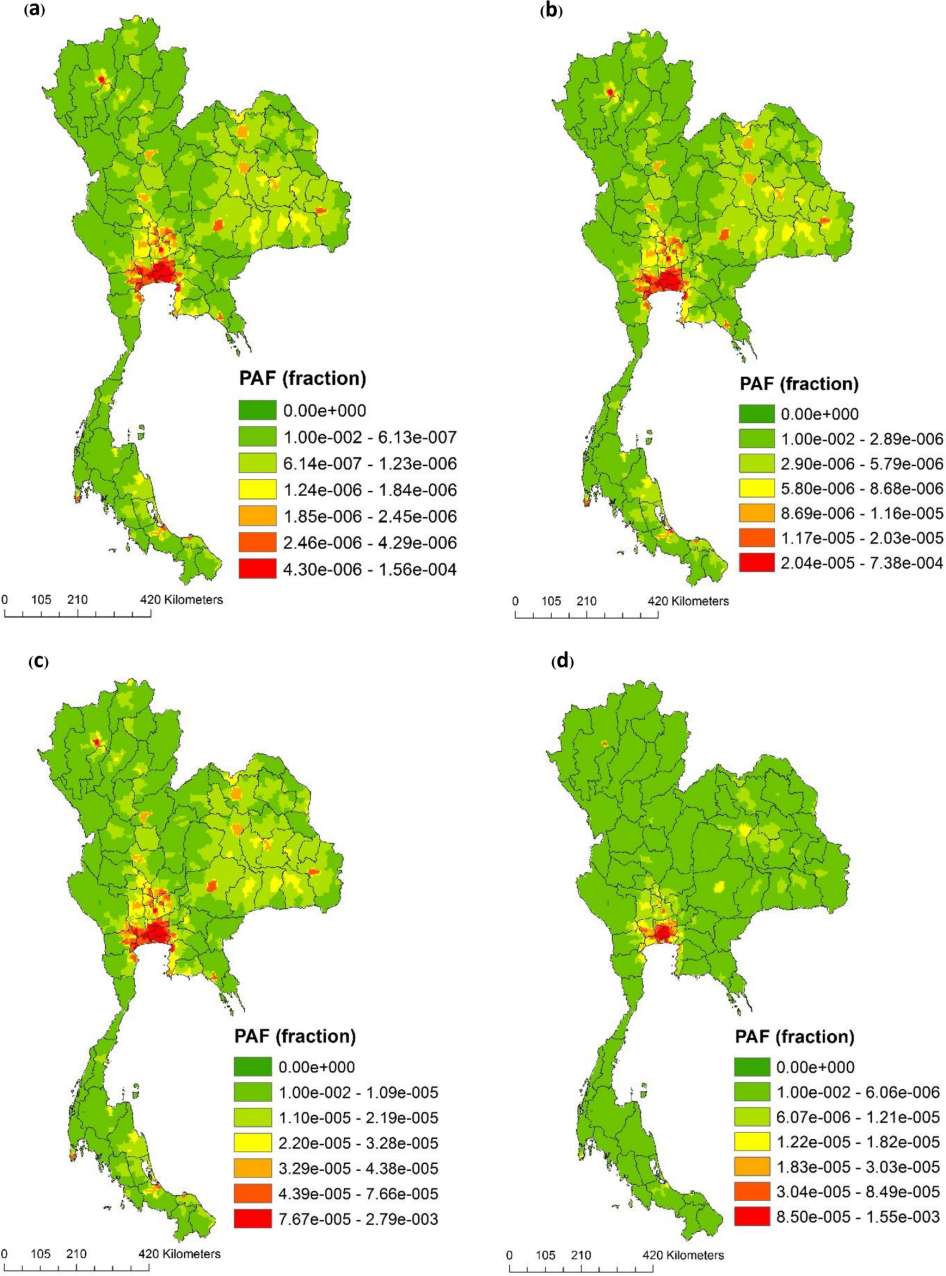
Spatial variations of population attributable fractions (PAF) in Thailand 2009. (a) All-cause mortality due to PM10, (b) all-cause mortality due to long- term effect of PM2.5, (c) cardiovascular mortality due to long- term effect of PM_2.5_ and, (d) respiratory mortality due to NO_2_.

This study estimated the average PAF grid for all-causes mortality due to PM_10_, PM_2.5_, NO_2_ at approximately 1.41 x 10^−6^ (95% CI: 1.35 x 10^−6^–1.47 x 10^−6^), 3.31 x 10^−6^ (95% CI: 3.17 x 10^−6^–3.45 x 10^−6^), and 4.6 x 10^−6^ (95% CI: 4.2 x 10^−7^–4.8 x 10^−6^), respectively. The average PAF for lung cancer caused by PM_2.5_ was approximately 8.16 x 10^−6^ (95% CI: 7.8 x 10^−6^–8.5 x 10^−6^). Fig 5 illustrates that the spatial variability of PAF due to long-term ambient air pollution exposure varied across Thailand. The results of this study indicated that the Bangkok Metropolitan Area had the largest percentage of total mortality attributable to PM_2.5_ across all ages (level ranged widely from 2.93 x 10^−4^ to 7.4 x 10^−4^ depending on the risk estimate used), which was the highest among the three air pollutants. The largest percentage of mortality attributable to NO_2_ was also the highest in the Bangkok Metropolitan Area (PAF ranged between 1.67 x 10^−4^ to 4.51 x 10^−4^ depending on the selected health end-point).

The summation of the PAFs for all grids in the category of air pollutants and disease outcomes based on Eq 2 and Table 1 are represented in Table 3. The PAFs for all-causes mortality for PM_10_, PM_2.5_, and NO_2_ were approximately 0.02, 0.1 and 0.1, respectively. The PAFs for respiratory mortality caused by PM_10_ and NO_2_ were approximately 0.02 and 0.07, respectively; PAFs for cardiovascular mortality caused by PM_10_ and PM_2.5_ were approximately 0.04 and 0.15, respectively, while PAF for lung cancer caused by PM_2.5_ was approximately 0.17. PM_2.5_ had the highest model estimated PAF at 17% of lung cancer burden.

Table 4 indicates the results for mortality caused by ambient air pollutants. The best estimate demonstrated ambient air pollution-related mortality, which included pollution from PM_2.5_, PM_10_ and NO_2_. Annually, there are about 3652–38410, 653–934 and 4024–15361 cases of all-causes, respiratory and cardiovascular mortality, respectively, from long-term exposure to PM and NO_2_, reflecting the highest CR from PM and the underlying cause-specific mortality for each health outcome.

**Table 4:** Avoidable mortality and the percentage of deaths estimated to be caused by level of air pollutant in Thailand (2009).

Pollutants Health outcome		Mortality, in hundreds			
		Current estimate (1)	Decrease 20% of pollutants concentration (2)	Avoided mortality (1–2)	Percentage of current estimate
**PM**_**10**_	All-cause	126.9	96.2	30.8	24.3
	Respiratory^a^	6.52	4.9	1.6	24.4
	Cardiovascular^a^	40.24	30.5	9.8	24.2
**PM**_**2.5**_	All-cause	269.9	210.1	59.8	22.2
	Cardiovascular^a^	153.6	11.9	34.0	22.1
	Lung-cancer^a^	24.6	19.1	5.4	22.0
**NO**_**2**_	All-cause	36.5	29.2	7.3	19.9
	Respiratory^a^	9.3	7.5	1.9	19.8

a The mortality numbers are not additive because these health outcomes are subsets of all-cause mortality.

The results in this study indicated that, if PM and NO_2_ were reduced by 20% from current levels, the health burden could be reduced in about 5982 cases for all-causes mortality, 160–581 and 146–3401 cases for respiratory and cardiovascular mortality, respectively, depending on each pollutant in Thailand. Similarly, the health burden would have been reduced annually to about 3081 cases for all-causes mortality per year if the highest concentrations for ambient particulate matter (PM_10_) across the country, which was about 84.6 μg/m^3^, had been reduced to 65 μg/m^3^. Respiratory mortality attributed to PM_10_ could be reduced annually to about 160 cases per year, or about 24.4% of the current estimate due to respiratory mortality. Other health outcomes of particulate matter, such as cardiovascular and lung cancer, could also be reduced annually to about 146 and 542 cases per year, respectively.

## Discussion

We presented a combination of GIS spatial analysis and empirical information on CRA to quantify the geographical distribution of PAFs and 2009 mortality due to various ambient air pollutants (PM_2.5_, PM_10_ and NO_2_) across Thailand, based on available empirical data. We predicted mortality attributable to short- and long-term ambient air pollution exposure ranging between 933 and 27 thousands persons depending on air pollutants across the country. PAFs varied across the country, as expected; PAF and exposure to air pollutants were relatively concentrated in the Bangkok metropolitan area, which had the largest number of monitoring stations, population density and air pollutant concentrations in Thailand, especially for PM and NO_2_.

Our estimate of all causes mortality attributable to PM_2.5_, was about 38,410 deaths or 6% of total deaths in Thailand. This proportion was not much different in terms of proportion compared to the GBD 2015[5], which had estimated 7.6% of total mortality for long-term exposure to PM_2.5_ globally. GBD used existing surface monitoring data to assemble a georeferenced global PM_2.5_ measurement database of 2005 annual average concentrations from available national/regional/local air quality monitoring reports and excluded PM_10_ and NO_2_ from their estimation [59]. The surface monitoring measurements dataset for the Asia region was based primarily on measured PM_2.5_ and appeared in the annual ambient air quality monitoring report from Australia and New Zealand [60]. The surface monitoring measurement datasets for other Asian countries (e.g. Thailand) were obtained from the Clean Air Initiative Asia (CAI-Asia) [59], which was generated from available datasets in 2005. All air quality stations were available for monitored important pollutants such as PM_10_ and NO_2_ in Thailand since the enhancement and conservation of the National Environmental Quality Act of 1992[61]. PM stations for the GBD study were about 16 stations for representing entire areas in the Southeast Asia region [59], reflecting significant evidence of air pollutant concentrations and long distance correlation (i.e. regional scale).

PAF is an estimation of the proportion of cases in the entire study population that can be attributed to air pollution. It can illustrate the health impact gained if the exposure to the counterfactual level can be reduced. The PAFs in this study were between 7.6% and 16.9% (Table 3) depending on health outcomes. In another approach to assess exposure based on the same RR information [3], Fann et al.[62] estimated that the largest percentage (between 7% and 17% depending on the health outcomes) of mortality attributable to PM_2.5_ was in southern California in the United States, using the Community Multiscale Air Quality (CMAQ) Modelling System[63] and health impact function. Anenberg et al.[20] also estimated the global burden of mortality due to PM_2.5_ to be about 2% for cardiopulmonary and lung cancer mortality, and 7% for all-causes mortality using the global atmospheric chemical transport model [64]. In another related study based on the same CR[3, 65], Ying Li et al. 2010[66] estimated the disease burden attributed to particulate matter exposure in the United Arab Emirates (UAE) from surface air monitoring station data and the spatial interpolated modelling technique. Their estimates of attributable fractions for PM were represented spatially and ranged from 12% to 28% of the total all-causes mortality in the UAE in adults aged >30 years in 2007, or at approximately 545 excess deaths annually[66]. The all-causes mortality due to PAF of PM_10_ and PM_2.5_ in our study were approximately 3 and 8%and lower compared to Ying Li et al. The means of PM_10_ concentration (μgm^-3^) for the UAE (90–665 μgm^-3^) were higher than those in Thailand (ranging between 20 and 84 μgm^-3^) because the UAE is situated in a desert region and severe dust storms occur in the Arabian Gulf region[66]. Moreover, the dispersion of pollutants from other continents may be another factor producing a high natural background of pollutants (e.g. PM_10_ 90 μgm^-3^ and PM_2.5_, 45 μgm^-3^).

According to a previous study on the mortality risk estimation due to air pollution in Thailand, the pollution mix, seasonality and demographics may be different from developed countries in Europe and North America[31]. We attempted to use available RR information from local epidemiological studies to reduce the bias caused by extrapolation of findings to another location[66]. Therefore, we used the RR information for PM_10_ all-cause and respiratory outcome from a local study that investigated the association between effects of exposure to air pollution on mortality risks in Thailand[9]. For NO_2_, we estimated all-causes mortality and respiratory mortality based on evidences from a systematic review and meta-analysis on a global scale due to the lack of RR information at the local level. However, the burden of NO_2_ showed the largest mortality contribution, and the high correlation between NO_2_ and PM_2.5_ (around 0.7–0.8) of meta-analysis still suggests the possibility that NO_2_ effects could be due in part to confounding from particulate matter. Hence, future epidemiological studies about information on the RR for Thailand should be conducted to reduce bias and improve PAF estimation.

Our results may be underestimated, since GBD recommended O_3_ as one of the indicators to quantify air pollution exposure associated with adverse health outcomes similar to those induced by PM (i.e. respiratory, cardiopulmonary diseases) [46, 66]. Several studies[67, 68] indicated that NO_2_ contributed O_3_ formation as a precursor with heavy traffic load, large population density and meteorological factors [69–71]. Moreover, mixtures of O_3_ and NO_2_ might react to form dinitrogen pentoxide (N_2_O_5_), that could create a greater risk than either O_3_ or NO_2_. Further studies should consider the analysis with the role of O_3_ as a possible important effect on the health outcomes.

For the quality of information on the levels of mortality and causes of death, several studies stated that mortality statistics in Thailand were low quality, with 20–40% of deaths are registered with unknown or nonspecific causes in the past decade [56, 72, 73]. However, we used the best available mortality information from the study that had been initiated to verify cause of deaths (COD) reported by vital registration from the nation-wide VA study[53, 56, 74], and adjusted the completeness of the vital registration was based on the mid census SPC conducted by the National Statistical Office [57, 58].

Our study might have some limitations and uncertainties. For the exposure assessment based on air quality monitoring station may depend on the location, density and distance of the monitoring network to nearby emission sources. In particular, the low number of measuring sites displayed in some regions (e.g. two and five stations in the north-eastern (about 160,000 km^2^) and southern (about 70,713 km^2^) regions, which may have some limitations in simulating the uniformly distributed annual ambient air pollution exposure on a large scale (e.g. national or regional scale. We recommend that empirical-based models at the national level are required to identify the priority sites of where new monitoring stations should be located to increase the air monitoring stations in a large population area [75], to improve the empirical-based estimation in future research.

Furthermore, monitoring stations with a measurement capacity for PM_2.5_ remained limited at the national level at the time of this study [76]. Therefore, we recommend the use of a PM_2.5_ /PM_10_ ratio based on available local study and empirical information in WHO’s EBD study[19] to estimate the exposure to PM_2.5_. As the remote sensing technique was used for estimating the surface PM_2.5_ concentrations from satellite observations, remote sensing-derived PM_2.5_ measures have been found to be well correlated with actual ground-level PM_2.5_ measurements [77, 78]. Therefore, future research may consider remote sensing data combined with ground monitoring station data in Thailand for greater precision in assessing PM_2.5_ exposure [47].

Another limitation was regarding the PAF estimation on a spatial scale based on two different grid resolutions of Pe and air pollutant concentration layer. Although, we used the resampling and interpolation technique in the raster algebra process[79], this may have produced some variation of grid and uncertainty from the estimation[66]. Further studies should employ a multi-spatial resolution approach [80, 81] and/or consider the consistency of spatial resolution on air concentrations and the population of exposure distribution [48].

Finally, our findings indicate a significant health impact due to air pollution problems in Thailand and that a 20% reduction in air pollutants could reduce the number of annual deaths by about 160–7,425 per year. Therefore, the government should increase its effort and investment into controlling air pollution to achieve the SDGs. As previously stated, air pollution problems and their burden of disease are geographically specific. Thus, autonomy and the capacities of local authorities in managing their own problems are certainly required, as well as a national healthy public policy framework [82] to effectively deal with these problems.

## Conclusions

This study aimed to quantify the magnitude and distribution of disease burden caused by ambient air pollution for policy-makers and planner by presenting an integrated exposure assessment, using a spatial interpolation model from empirical data, population distribution exposure and health impact function to estimate the national disease burden attributable to ambient air pollution. In addition, the GIS-based population exposure assessments for PAFs and the estimation of the number of deaths due to ambient air pollution exposure are useful for prioritising policy to reduce and prevent adverse health effects in Thailand. We hope that our findings offer a national estimate and benefit decision-making by stakeholders and policy-makers to promote and develop air quality management and health co-benefit strategies to achieve the SDGs in the future.
